# Prostatic artery from an extrapelvic obturator artery: a rare common femoral artery variant with clinical implications

**DOI:** 10.1186/s42155-025-00594-3

**Published:** 2025-10-11

**Authors:** Omar Ayman, Paul Bennett Lewis

**Affiliations:** Department of Radiology, College of Medicine, The Ohio State University Wexner Medical Center, 432 12th Street, Suite 426, Columbus, OH 43210 USA

**Keywords:** Prostatic artery embolization, Aberrant obturator artery, Prostatic artery, Interventional radiology, Variant anatomy, Embolization, BPH

## Abstract

**Background:**

Prostate artery embolization (PAE) is an established treatment option for benign prostatic hyperplasia (BPH). Variations in prostatic artery (PA) origins can present significant technical challenges.

**Case presentation:**

An 86-year-old male with recurrent bladder cancer and persistent gross hematuria post-TURBT presented for PAE. Intra-procedural angiography revealed a prostatic artery branching from an aberrant obturator artery that originated from a trifurcation of the common femoral artery. PAE was successfully performed with contralateral access and particle embolization. The patient’s hematuria resolved within 3 days, and his IPSS decreased by 10 points at follow-up.

**Conclusion:**

This case highlights a markedly rare variant of the prostatic artery arising from the common femoral artery, emphasizing the need for careful pre-procedural planning and vigilance during PAE to avoid complications.

**Supplementary Information:**

The online version contains supplementary material available at 10.1186/s42155-025-00594-3.

## Background

Prostate artery embolization (PAE) is now a well-established therapeutic option for benign prostatic hyperplasia (BPH) [1]. However, the marked variability in prostatic artery (PA) origins can present significant technical challenges to successful treatment. This case report details a prostatic artery originating from trifurcation of the common femoral artery into the superficial femoral, profunda femoris, and a replaced obturator artery. Origin of a PA from an obturator artery originating from the common femoral artery has not been previously encountered. Institutional Review Board (IRB) approval was not required for this case report. Informed consent was obtained for the publication of this case.

## Case presentation

An 86-year-old male with recurrent bladder cancer was admitted for persistent gross hematuria 29 days after his third transurethral resection of bladder tumor (TURBT). His surgical history also included one prior transurethral resection of prostate (TURP) but no open procedures. On his second hospital day, urology performed a cystoscopy with TURBT and TURP. Management of hematuria then began with continuous bladder irrigation and blood product transfusion. His bleeding was recalcitrant to these conservative measures and he was referred to IR for PAE on hospital day 7. His prostate gland was 139 mL and his International Prostate Symptoms Score (IPSS), 22.

The pre-procedural computed tomography angiography (CTA) was limited by bolus timing. The bilateral PA origins were poorly-defined but presumed to be conventional in nature. The patient provided his informed consent for bilateral pelvic angiogram with possible prostatic artery embolization.

**Fig. 1:**
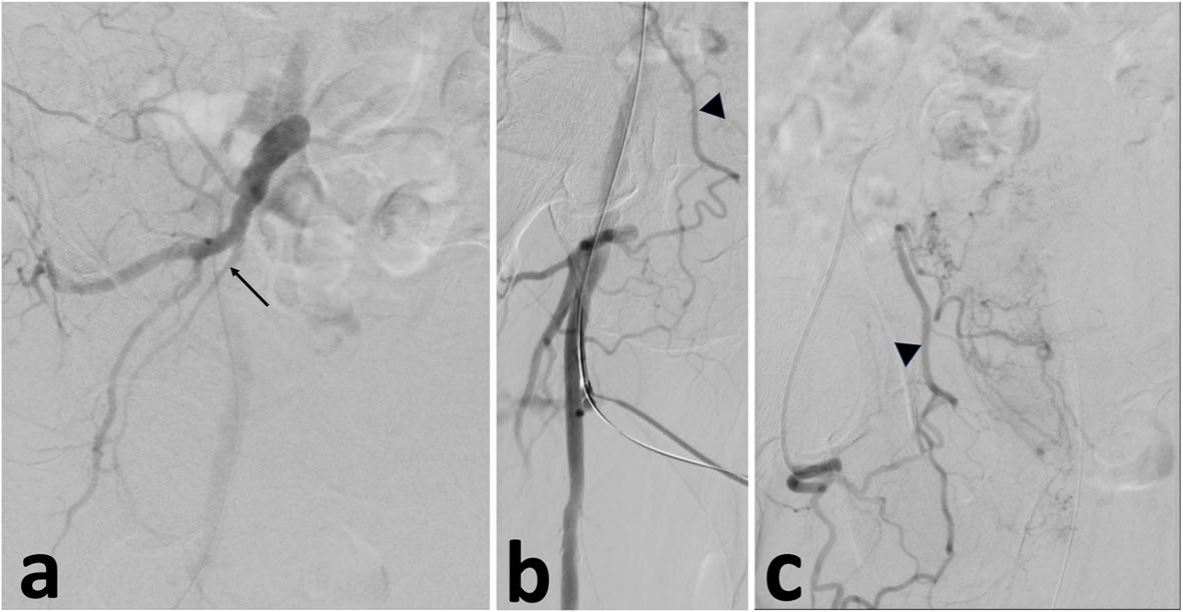
Intra-procedural digitally subtracted angiograms (DSA). **a** Right anterior oblique DSA from the right internal iliac artery opacifies the posterior division arteries and the internal pudendal artery (arrow) but no superior vesical or prostatic artery. **b** Initial right anterior oblique of the right common femoral artery DSA after obtaining access opacifying the cranially-directed branches reconstituting the obturator artery (arrowhead). **c** Anterior–posterior selective DSA of the common trunk of the cranially-directed branches from contralateral access subsequently opacifying the obturator artery (arrowhead) and right half of the prostate

The right common femoral artery was accessed with a 5 Fr 10 cm Pinnacle sheath (Terumo, Tokyo, Japan) and attention was first directed to the left prostatic artery, which had a Type III origin [2]. Straightforward embolization of the left prostatic artery was performed with 100–300 um Embosphere particles (Merit Medical Systems, South Jordan, Utah, USA) through a 4.8-French Progreat® microcatheter (Terumo, Tokyo, Japan) and a 5 French IMPRESS® C2 Cobra (Merit Medical, Inc., South Jordan, UT, USA). After selecting the right internal iliac artery with a SOS 1 catheter (AngioDynamics, Latham, New York, USA), the angiogram demonstrated a conventional bifurcation to anterior and posterior divisions (Fig. 1a). The internal iliac artery had an internal pudendal artery but lacked a superior vesical and prostatic artery (Fig. 1a). Review of the initial right common femoral arterial run revealed a cranially directed branch at the common femoral artery branching point (Fig. 1b). Further investigation found the common femoral artery trifurcated to the superficial femoral, profunda femoris and an aberrant obturator artery (AOA) that ultimately supplied the PA. Fluoroscopic confirmation of the common femoral trifurcation into the obturator, superficial femoral and profunda femoris arteries is provided in Additional File 1 (video clip).

In the case of an inferior epigastric artery take-off, the reverse curve catheter that was used to select the internal iliac artery could be redirected to select the inferior epigastric artery as well. Here, use of the reverse curve SOS 1 catheter (AngioDynamics, Latham, New York, USA) was not attempted because the take-off was too low (i.e., downstream) from the site of access. Accordingly, the left common femoral artery was accessed.

Antegrade angiography of the right pelvis through the C2 Cobra catheter confirmed the common femoral artery trifurcation that included an AOA. Take-off of this AOA was clearly distinct and downstream from the inferior epigastric artery and separate from the medial circumflex femoral artery. This replaced obturator artery later supplied the right PA (Fig. 1c). After super-selection of the right PA with a 4.8-French Progreat® microcatheter and a Synchro microwire (Stryker Neurovascular, Fremont, CA, USA), an angiographic run confirmed the perfusion territory and embolization was then performed with a vial of 100–300 um Embospheres. The bilateral groin access was closed using Mynx closure devices (Cordis, Miami Lakes, Florida, USA) and the patient returned to the clinical floor.

**Fig. 2:**
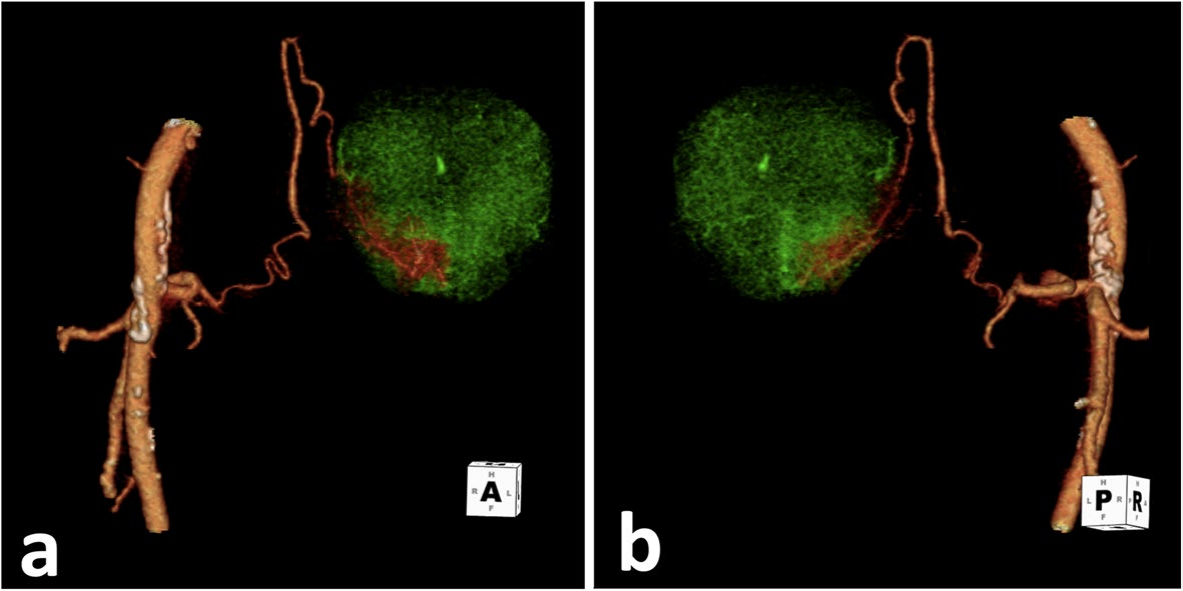
3-D volume-rendered technique (VRT) reconstruction from the pre-procedure CTA of the prostatic artery origin (**a**) Anterior view of the aberrant prostatic artery. **b** Posterior view of the trifurcation giving rise to the prostatic artery

The patient’s hematuria resolved within 3 days after PAE. At six-week follow-up, his clinical improvement was sustained with a 10-point reduction of his IPSS to a 12. The pre-procedure CTA was reconstructed with angiographic images as a guide (Figs. 2a and b).

## Conclusions

This case report presents an extremely rare anatomical variation where the PA originated from the common femoral artery via a trifurcation into a replaced obturator, superficial femoral and profunda femoris artery. The sole common feature in this case is its occurrence on the right side, as pelvic vasculature is more commonly variable on the right side than the left [3].

The rarity of this case is highlighted when viewed in the context of the other aberrant origins of obturator and/or prostatic artery. Approximately one-third of AOA arise from the inferior epigastric artery or the external iliac artery [3, 4]. The incidence of a PA originating from an AOA from either of those more common aberrant locations is < 2% [5]. An AOA from the common femoral artery was found 2.5% of the time in a 2023 systemic review and meta-analysis of 25 studies encompassing 3,453 hemi-pelvises by Sume and Mulu [3]. There is no direct reported prevalence of an obturator artery originating from the CFA and giving off the PA. Using combined reported rates from de Assis [2] and Sume and Mulu [3] gives an estimated prevalence of 0.47%, which likely grossly overstates the true prevalence due to methodological and population differences of the studies. Overall, this anatomy is likely a less common corona mortis variant.

Corona mortis variants represent vascular anastomoses between the obturator artery and an extrapelvic source: most commonly the inferior epigastric artery, external iliac artery, or femoral artery. Another rare corona mortis variant – and the most comparable to this case – is a prostatic artery arising from the medial circumflex femoral artery [6].

The vessel supplying the PA in this case was identified as an AOA. However, the possibility that it represents a retrograde collateralization due to proximal occlusion of a native obturator artery cannot be definitively excluded. That said; there was no angiographic evidence of proximal stenosis or occlusion, nor were there other collateral pathways observed. Additionally, the contralateral pelvic vasculature demonstrated no significant atherosclerotic disease.

While a pre-procedure CTA is not perfect [7], this case underscores its utility in procedural planning. In this case, CTA interpretation was limited by suboptimal bolus timing, which inhibited confident identification of the variant origin. Hindsight guided by angiographic findings allowed recognition of the atypical anatomy in the CTA. The origin was identifiable angiographically but remarkably rare.

In cases the prostatic artery is not identified from the anterior division of the internal iliac artery, first reassess imaging technique (e.g., bolus volume, injection rate, image intensifier angle) and review any available pre-procedure imaging. Perform a repeat run after making the necessary adjustments, including optional nitroglycerin administration. If still not visualized, perform a less selective internal iliac run including both divisions, followed—if needed—by a common iliac or selective external iliac run. The field of view in the external iliac run should extend inferiorly enough to include the common femoral artery. If the PA is still not identified, the final step is a cone-beam CT (CBCT). Here, plain fluoroscopy was sufficient for perfusion confirmation and CBCT was not required.

Corona mortis–like anatomy should be regarded as a serious procedural hazard. The aberrant connection between pelvic and extrapelvic vessels, the anomaly establishes an unanticipated route into the lower extremity. During embolization, this pathway amplifies the risk of devastating non-target ischemia, including catastrophic limb-threatening complications.

With growing adoption, PAE continues to gain worldwide acceptance and operators must remain vigilant to variant PA origins – notably extrapelvic origins – and be adaptable to them to control operative times, limit radiation doses, mitigate complications and improve procedural success rates. This case highlights that need to adjust when there is anatomic deviation from normal expectations.

## Supplementary Information

Additional file 1: Fluoroscopic run demonstrating the extrapelvic origin of the AOA from the right common femoral artery trifurcation (MP4 digital video file; 8 s).

## Data Availability

Not applicable.

## Abbreviations

PAE: Prostate artery embolization
BPH: Benign Prostatic hyperplasia
PA: Prostatic artery
IRB: Institutional Review Board
TURBT: Transurethral resection of bladder tumor
TURP: Transurethral resection of prostate
IPSS: International Prostate Symptoms Score
CTA: Computed tomography angiography
AOA: Aberrant obturator artery
